# Development of a Novel Cysteine Sulfinic Acid Decarboxylase Knockout Mouse: Dietary Taurine Reduces Neonatal Mortality

**DOI:** 10.1155/2014/346809

**Published:** 2014-02-03

**Authors:** Eunkyue Park, Seung Yong Park, Carl Dobkin, Georgia Schuller-Levis

**Affiliations:** ^1^Laboratory of Cellular Immunology, Department of Developmental Neurobiology, New York State Institute for Basic Research in Developmental Disabilities, Staten Island, NY 10314, USA; ^2^College of Veterinary Medicine, Konkuk University, Seoul 143-701, Republic of Korea; ^3^Laboratory of Molecular Genetics, Department of Human Genetics, New York State Institute for Basic Research in Developmental Disabilities, Staten Island, NY 10314, USA

## Abstract

We engineered a CSAD KO mouse to investigate the physiological roles of taurine. The disruption of the CSAD gene was verified by Southern, Northern, and Western blotting. HPLC indicated an 83% decrease of taurine concentration in the plasma of CSAD^−/−^. Although CSAD^−/−^ generation (G)1 and G2 survived, offspring from G2 CSAD^−/−^ had low brain and liver taurine concentrations and most died within 24 hrs of birth. Taurine concentrations in G3 CSAD^−/−^ born from G2 CSAD^−/−^ treated with taurine in the drinking water were restored and survival rates of G3 CSAD^−/−^ increased from 15% to 92%. The mRNA expression of CDO, ADO, and TauT was not different in CSAD^−/−^ compared to WT and CSAD mRNA was not expressed in CSAD^−/−^. Expression of Gpx 1 and 3 was increased significantly in CSAD^−/−^ and restored to normal levels with taurine supplementation. Lactoferrin and the prolactin receptor were significantly decreased in CSAD^−/−^. The prolactin receptor was restored with taurine supplementation. These data indicated that CSAD KO is a good model for studying the effects of taurine deficiency and its treatment with taurine supplementation.

## 1. Introduction

Taurine (2-aminoethanesulfonic acid), which is essential during the development of mammals, is not incorporated into proteins [1]. It is mainly produced in the liver and kidney and is present in most other tissues including the brain, leukocytes, retina, heart, placenta, and muscle [1–3]. Taurine is a key element in many diverse processes including development of the brain, retina, and the immune system, osmoregulation, reproduction, membrane stabilization, regulation of cardiac muscle, and inflammation [2, 4–10]. Colostrum contains a very high taurine concentration which is required for development of the brain and retina in the newborn human [11]. In response to research findings, taurine is universally added to infant formula as well as to parenteral solutions [2, 12].

The biosynthesis of taurine from methionine or cysteine can occur by two distinct pathways. Cysteine is oxidized by cysteine dioxygenase (CDO; EC 1.13.11, MW 24 kD) to cysteine sulfinic acid which is converted by CSAD to hypotaurine which is then oxidized to taurine. CSAD (EC 4.1.1.29, MW 51 kD) is a cytosolic enzyme expressed primarily in liver and kidney [13–16]. The enzyme responsible for the alternative pathway for taurine biosynthesis is ADO (EC 1.13.11.19, MW 28 kD) [17, 18]. Cysteine is incorporated into coenzyme A (CoA), followed by the release of cysteamine during CoA turnover. Cysteamine is oxidized to hypotaurine by ADO. Hepatic CSAD and CDO activity is high compared to that in the kidney and brain [15, 19–21]. The tissue distribution of CSAD, protein, and mRNA, detected by Western and Northern blot analyses, is consistent with CSAD activity. ADO level is highest in the brain, whereas CSAD and CDO levels are highest in the liver [15, 17, 18, 22].

Cysteine sulfinic acid decarboxylase (CSAD) is one of rate-limiting enzymes for taurine biosynthesis [2, 13] and the level of its activity determines the need for dietary taurine. Cats have been used for taurine studies because they have low levels of CDO and CSAD leading to a dependence on dietary sources of taurine. Rodents have high levels of CSAD [1, 2, 23] and taurine is not essential to their diet. Taurine is considered a conditionally essential amino acid in humans and is required in their diet at certain times during development. Analysis of murine CSAD cDNA shows that the enzyme has 98% and 90% homology to rat and human CSAD, respectively [14, 24]. Since the cat model has limitations including a long gestation period, a heterogeneous genetic background, and a relatively large maintenance expense, we developed a CSAD knockout mouse (CSAD KO) model to better understand the physiological roles of taurine. This novel murine model was developed to provide insight into the role of taurine in reproduction, innate and adaptive immunity, and brain development. We report here that the absence of a functional CSAD gene in the CSAD KO mouse reduces the level of this amino acid by >80% and has a severe effect on neonatal survival that is reversed by adding taurine (0.05%) to the drinking water.

## 2. Materials and Methods

### 2.1. Materials

Chemicals used in this study were purchased from Sigma Chemicals (St. Louise, MO) if not otherwise noted. Oligonucleotide primers for PCR were obtained from Eurofins MWG Operon (Huntsville, AL). Primers were designed by Primer Designer 4 (Scientific and Educational Software, Cary, NC). Taq polymerase and deoxynucleotides were obtained from New England Biolabs (Ipswich, MA). Agarose was obtained from Lonza Group LTD (Rockland, ME). Nitrocellulose membranes for Western blot and nylon membranes for Northern blot were purchased from Invitrogen (Carlsbad, CA) and NEN Life Science Products (Boston, MA), respectively. Membranes for Southern blot were obtained from Bio-Rad (Hercules, CA). Trizol and RNeasy kit for RNA extraction and DNeasy kit for DNA extraction were obtained from Invitrogen and Qiagen (Valencia, CA), respectively. Protease inhibitors were purchased from Roche Applied Science (Indianapolis, IN). Restriction enzymes were purchased from Promega (Madison, WI). [^32^P]-dCTP was supplied by Perkin Elmer (Waltham, MA).

### 2.2. CSAD KO Mice

Chimeric CSAD KO mice were produced by injection of cells from a gene trap ES cell line (XP0392) into C57BL/6 (B6) blastocysts at the Mouse Mutant Regional Resource Centers (MMRRC, UC Davis, CA) which were implanted into a pseudopregnant B6 mouse. Nine chimeric mice from MMRRC were mated with B6 (Jackson Laboratories, Bar Harbor, ME) in the animal colony at this institution (NYS Institute for Basic Research in Developmental Disabilities). Agouti mice produced by this mating were back-crossed to B6 and offspring were genotyped using PCR to identify germline transmitted CSAD^+/−^ heterozygotes (CSAD^+/−^). Heterozygous siblings were mated to produce CSAD^−/−^ homozygous pups (HO). Mice were fed taurine-free chow (LabDiet, PMI Nutrition International, St. Louis, MO). Taurine concentrations in commercial food were confirmed by HPLC. All mice were kept under 12 hr day/night with free access to food and water. Some experimental animals had exogenous taurine added to their water at 0.05% as indicated in Section 3. For optimum reproductive performance, one or two females were mated to a single male. Both females and males used for mating in the taurine-treated groups were supplemented with taurine. Animals were weaned at 3 to 4 weaks of age.

All procedures involving live animals were approved by the Institutional Animal Care and Use Committee of IBR.

### 2.3. Southern Blot Analysis

DNA from the spleen was analyzed by Southern blotting to confirm the CSAD KO genotype using a modified method previously described [25]. DNA from the spleen was prepared with DNeasy kits (Qiagen, Valencia, CA). DNA was digested with EcoR V (Promega), fractionated by agarose gel electrophoresis, and transferred to a nylon membrane (Bio-Rad). The membrane was hybridized using CSAD cDNA as a probe to confirm the disruption of the CSAD gene. CSAD cDNA was prepared as previously described [14].

### 2.4. Genotype Determination

Template DNA was isolated from a tail sample at 3 weeks of age with a DNeasy kit (Qiagen) and analyzed with two sets of primers, *β*-geo and vector insertion site (VIS) (Table 1), using a PCR kit (Perkin Elmer, Shelton, CT). After an initial of 93°C for 3 min, DNA was denatured at 93°C for 1 min, annealed at 63°C for 1 min, and extended at 72°C for one and a half min for 30 cycles. DNA was extended for additional 5 min. PCR products were characterized by agarose gel electrophoresis. The sizes of PCR products using *β*-geo and VIS primers were 682 bp and 1038 bp, respectively. DNA from HO and CSAD^+/+^ wild type (WT) produced one band from either *β*-geo or VIS, respectively, and CSAD^+/−^ produced bands from both *β*-geo and VIS.

### 2.5. Northern Blot Analysis

Northern blot analysis was performed as previously described [10]. Briefly, total RNAs from liquid nitrogen frozen liver were extracted with Trizol and RNeasy kit. RNA was separated by agarose gel electrophoresis and transferred to a nylon membrane (NEN Life Science Product). The membrane was hybridized using [^32^P]-dCTP labeled CSAD cDNA as probe.

### 2.6. Western Blot Analysis

Western blot analysis was performed according to the method previously described [10]. Briefly, frozen liver and brain were homogenized with PBS buffer containing IGEPAL CA-630 and protease inhibitors (Roche Applied Science). Supernatants from tissue homogenates were separated using SDS-PAGE and transferred to a nitrocellulose membrane. The membrane was incubated with rabbit anti-CSAD antibody and then with alkaline phosphatase labeled goat anti-rabbit secondary antibody after washing the membrane with PBS containing 0.2% Tween 20. The membrane was visualized using 5-bromo-4-chloro-3-indolyl phosphate/nitroblue tetrazolium. Polyclonal rabbit anti-CSAD was produced in our laboratory using c-terminal 15 peptides obtained from Synpep Corp. (Doblin, CA) [14].

### 2.7. High Performance Liquid Chromatography (HPLC)

The levels of taurine were determined using HPLC (Waters, Milford, MA) [26]. Briefly, tissues were homogenized using 5% TCA and centrifuged for removal of proteins. After samples were dried using a Speedvac (Savant, Holbrook, NY), they were derivatized using phenylisothiocyanate (PITC) and separated using a C18 column with a gradient of acetate buffer containing 2.5% acetonitrile (pH 6.5) and 45% acetonitrile solution containing 15% methanol at 45°C. The flow rate was 1 mL/min. Taurine concentrations were determined by comparison to a standard.

### 2.8. Microarray Analysis

Total RNAs from the brain and liver in WT and G3 HO at postnatal day 1 (PD1) were extracted using RNeasy mini kit (Qiagen) according to manufacturer's instructions. Biotin-RNA was generated using the Epicentre Target Amp-Nano Labeling Kit for Illumina Expression BeadChip (Cat number TAN07908, San Diego, CA) from an input of 300 ng of high quality RNA. Illumina bead arrays were performed using Mouse WG-6 v2 Bead Chip (Cat number BD-103-0204) and Illumina gene expression kit according to manufacturer's protocol. Raw intensity values were acquired using the HiScan microarray scanner (Illumina) and imported to Genome Studio using the Gene Expression Module (Illumina).

### 2.9. *RT* ^2^ qPCR Analysis

Total RNA was extracted using RNeasy kit (Qiagen) from the brain and liver at PD 1 and was reverse-transcribed using cDNA kit according to the manufacturer's instruction (Qiagen). Quantitative real time PCR with 10 ng of cDNA were carried out in duplicate in a 7300 real time PCR system (Effendorff, Hauppauge, NY) using the SYBR master mix (Qiagen) and the following cycles: 2 min at 50°C, 10 min at 95°C, and then 40 cycles each at 95°C for 15 s and 60°C for 60 s [19]. *RT* ^2^ qPCR analysis was also carried out according to manufacturer's manual using *β*-actin as a control. All primers used in this study were purchased from Qiagen. For data analysis the *Ct* method was used; for each gene fold changes were calculated as difference in gene expression of G3 HO and G3 HOT compared to that in WT. Δ*Ct* was calculated by subtraction of *Ct* of *β*-actin from *Ct* of the specific gene. ΔΔ*Ct* was calculated by subtraction of Δ*Ct* of WT from Δ*Ct* of G3 HO or H3 HOT. Fold change was determined by 2^ΔΔ*Ct*^. More than 1 indicates gene upregulation and less than 1 indicates gene downregulation.

### 2.10. Statistical Analysis

Data were represented as mean ± SE. Statistical significance was determined using Statistica 8 (StatSoft, Tulsa, OK). Significant differences between groups were determined as *P* < 0.05 using LSD or tukey HSD in post hoc under one way ANOVA.

## 3. Results

### 3.1. CSAD KO Mouse

**Figure 1:**
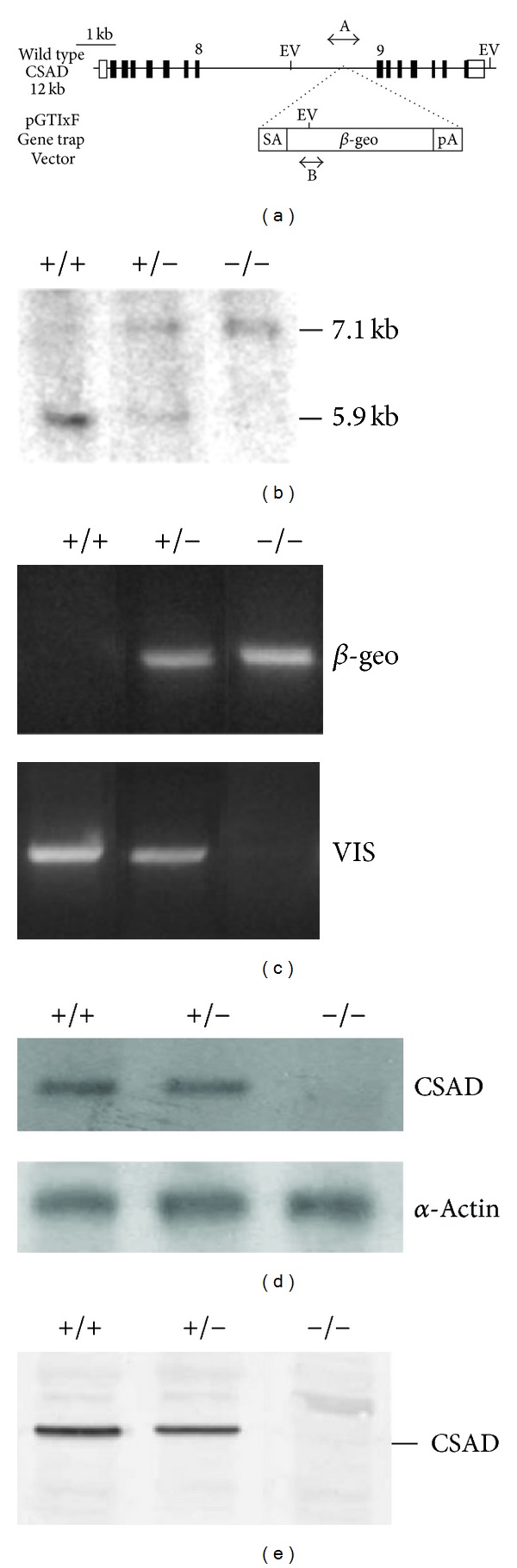
Analysis of the CSAD gene knockout. (a) Schematic diagram of pGT01xf insertion into the CSAD gene. Rectangles represent exons and open rectangles represent, untranslated regions. “A” shows the location of the VIS amplicon which is produced from the wild type chromosome and “B” shows the location of the *β*-geo amplicon which is produced from the gene trap vector inserted between exons 8 and 9. SA: splicing acceptor; pA: polyadenylation sequence; EV: EcoR V sites. (b) Southern analysis of genomic DNA digested with EcoR V and hybridized using [^32^P]-dCTP labeled CSAD cDNA. The wild type CSAD EcoR V fragment is 5.9 kb and that of disrupted fragment is 7.1 kb. “+/+”: WT; “+/−”: CSAD^+/−^; “−/−”: CSAD^−/−^. (c) PCR products (1038 bp and 682 bp) with VIS and *β*-geo primer sets. *β*-geo product is absent in WT and VIS PCR product is absent in CSAD^−/−^. Both PCR products are present in CSAD^+/−^. (d) Northern blot analysis of total RNA from liver with [^32^P]-dCTP labeled cDNA and *α*-actin as a control probe. (e) Western blot analysis of kidney homogenates probed with an anti-CSAD antibody and detected with an alkaline phosphatase labeled goat anti-rabbit antibody. CSAD (51 kD) was not detected in CSAD^−/−^ homogenates.

The murine CSAD gene is located on chromosome 15:102, 176, 998-102, 189, 043 bp (assembly Dec. 2011; GRCm38/mm10). Chimeric mice were derived from an ES cell line in which the CSAD gene had been inactivated by insertional mutagenesis (XP0392, MMRRC: 022638-UCD Sanger gene trap line) [Figure 1(a)]. ES cells were inserted into B6 blastocysts at the MMRRC [27, 28] (work supported by NCRR-NIH). Chimeric males (obtained from MMRRC) were mated with wild type B6 females. The genotypes of the resulting offspring were determined by PCR using tail DNA with two pairs of primers (Table 1). One primer pair (*β*-geo) detects the presence of the gene trap insertion (vector), located between exons 8 and 9 in the CSAD gene [Figures 1(a) and 1(c)], and the other primer pair (VIS) detects the wild type (WT) sequence at the insertion site and gives no product in the presence of the insertion. The sizes of the *β*-geo and VIS PCR products are 682 and 1038 bp, respectively. Southern blot analysis of homozygous CSAD KO mice (HO) showed disruption of the CSAD gene [7.1 kb in Figure 1(b)]. WT and CSAD^+/−^ showed one band (5.9 kb) and two bands (5.9 and 7.1 kb), respectively. The CSAD protein (51.0 kD) and CSAD mRNA were detected in WT and CSAD^+/−^ but were not detected in HO [Figures 1(e) and 1(d)]. Plasma was collected by heart puncture from all three groups at age of 8 weeks. Plasma taurine concentration, determined by HPLC, was significantly reduced in CSAD^+/−^ but was drastically reduced by more than 80% in G1 HO and G2 HO compared to WT (Table 2). Taurine concentrations were not significantly different in females compared to males and data from 8-week-old males and females were pooled. Taurine is not completely absent in HO presumably because there is an alternative pathway for taurine biosynthesis from cysteamine [17] through cysteamine dioxygenase (ADO). CSAD^+/−^ × CSAD^+/−^ mating produced offspring genotypes that were not different from the expected Mendelian distribution.

### 3.2. CSAD KO Reproduction

**Figure 2:**
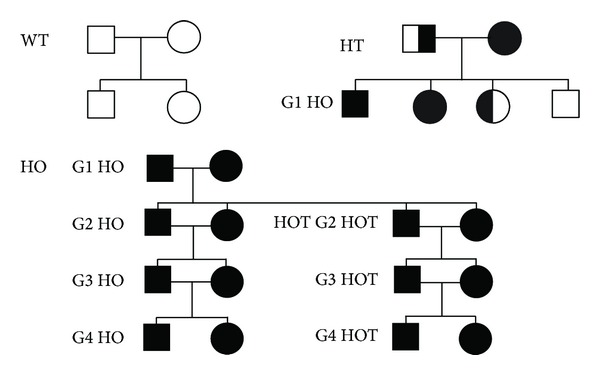
Mating scheme. G represents generation of mice and T for taurine treatment. Squares and circles represent male and female, respectively. White is WT, half black is HT, and black is HO; G2 HOT were treated with 0.05 % taurine in the drinking water 10 days prior to mating and G3 HOT were treated with taurine after weaning (3-4 weeks of age).

**Figure 3:**
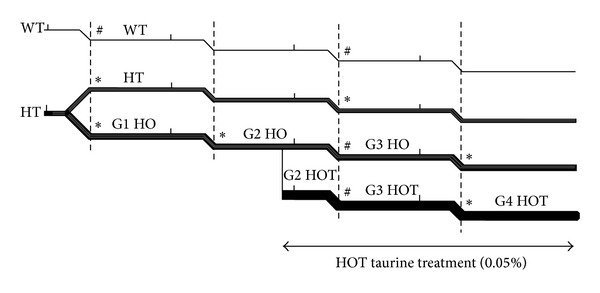
Schematic diagram for generation of different genotypes and sampling tissues for taurine concentrations, microarray analysis, and *RT* ^2^ qPCR. Generations of different genotypes are shown as stepped horizontal lines. Short vertical lines indicate mating of 8-week-old mice. Short diagonal lines indicate birth of pups. Taurine treatment of G2 HO animals (double headed arrow) began 10 days before mating. Dashed vertical lines locate PD1. Asterisks (∗) indicate observation of pup survival at PD1 and samples taken for taurine concentrations in the brain and liver. Hash marks (#) indicate observation of pup survival at PD1 and samples taken for taurine concentrations in the brain and/or liver as well as tissue samples taken at PD1 for microarray analysis and *RT* ^2^ qPCR of the liver and brain mRNA.

To investigate the role of taurine in reproduction, we observed three generations of homozygous CSAD^−/−^. Homozygotes are abbreviated as follows: G1 HO were born from CSAD^+/−^ × CSAD^+/−^; G2 HO were born from G1 HO × G1 HO; G3 HO were born from G2 HO × G2 HO (Figures 2 and 3). There were no significant differences from wild type (WT) in the pregnancy rates in different generations of HO mice. There was also no difference in the number of females and males that were bred and the number of pregnancies per female averaged 3-4 in all groups. In addition, the initial number of pups per litter did not vary significantly across groups (Table 3). Although the concentrations of taurine in the plasma were low in G1 HO (Table 2), all of the offspring from G1 HO pairs survived and thrived (Table 3). However, offspring from G2 HO and G3 HO had a very low rate of survival, although a normal number of pups were born alive. All of the pups from eleven out of thirteen litters (85%) born from G2 HO and from nine out of ten litters born from G3 HO (90%) were dead within 24 hrs (Table 3). Death/survival was measured at postnatal day one (PD1) (Figure 3). Survival rates of G3 and G4 HO litters born from G2 and G3 HO were 15% and 10%, respectively. The surviving pups all showed “milk spots” (white material in their stomachs visible through the skin) while there were no milk spots apparent in the pups that died. Birth weights of live HO including G3 HO and G4 HO and WT were not significantly different although G3 and G4 HO pups without milk spots were slightly smaller at PD1 (~0.1 g) compared to those with milk spots. Surviving pups from G2 HO and G3 HO grew normally although they were very few in number.

Since the surviving pups had evidence of successful nursing while the others did not, we cross-fostered HO pups with WT dams and WT pups with HO dams to see whether the fault lay in the dams nursing behavior or the pups' suckling. When WT neonates born were nursed by G2 HO dams (that had delivered pups the same day), they suckled milk and grew normally. Neonates without milk spots born from G2 HO did not survive when they were nursed by WT dams.

To see the effect of exogenous taurine we also observed three generations of homozygous CSAD^−/−^ that were treated with 0.05% taurine in their drinking water (HOT) (Figure 3). Both males and females of G2 HOT were treated with taurine (0.05%) in their drinking water from 10 days prior to mating until weaning of their offspring. G3 HOT were born from G2 HOT and treated with taurine from weaning to their offspring's weaning. Twelve of thirteen litters and fifteen of sixteen litters from G2 and G3 HOT, respectively, treated with 0.05% taurine in the drinking water thrived. The survival rates of litters from G2 HOT and G3 HOT were 92% and 94%, respectively. There was no statistically significant difference in reproduction performance between G2 HOT and G3 HOT; pregnancy rates and the number of pups per litter were not different from WT. Pups from WT, HT, HO, and HOT were weaned 3 to 4 weeks after birth. Growth of pups from WT, HT, and HOT was not significantly different. The high survival rates of litters from both taurine-supplemented groups indicated that proper levels of taurine during gestation are necessary for survival. The death of most pups in the nonsupplemented G2 and G3 HO litters within 24 hours of birth suggests that the lack of taurine *in utero* may have been the important factor.

### 3.3. Taurine Concentrations in the Liver and Brain

**Figure 4:**
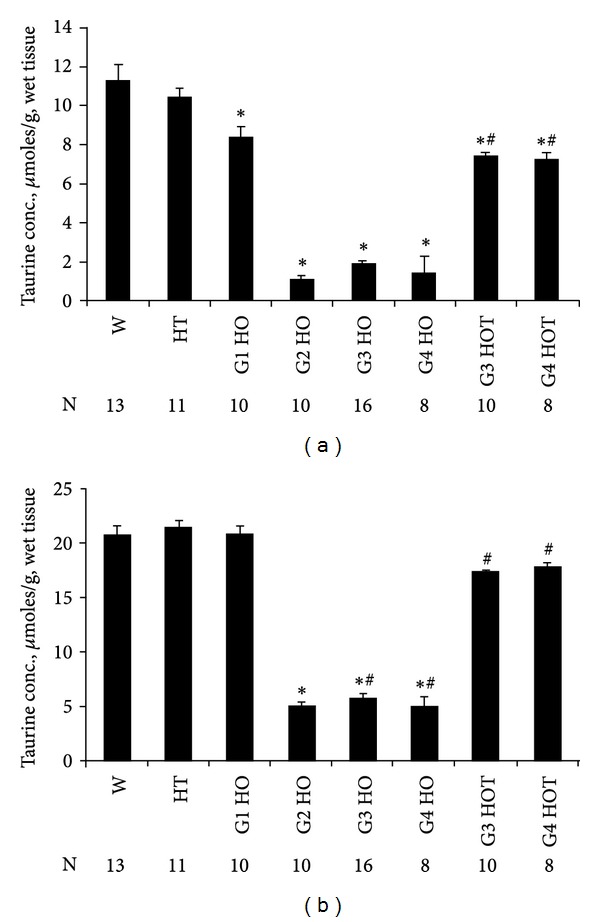
Taurine concentrations in the liver and brain. The livers (a) and brains (b) at PD1 in each group were homogenized with 5% TCA and centrifuged at 10,000 ×g for 20 min. The supernatants were collected and dried using a Speedvac. Dried samples were derivatized using PITC. PITC labeled taurine was determined using HPLC. Data are expressed as *μ*moles/g wet tissue weight, mean ± SE in (a) the liver and (b) the brain. *P < 0.002 is statistically significant, compared to WT. ^#^ *P* < 0.001 is statistically significant, compared to G3 HO and G4 HO.

Since the liver is a major source for the production of taurine required for brain development [2], taurine concentrations in both liver and brain were measured at PD1 [Figures 3, 4(a) and 4(b)]. Taurine concentrations in the brain and liver were significantly reduced by more than 80% in G2 HO, G3 HO, and G4 HO but not in G1 HO in the brain which were born from heterozygote dams with normal levels of taurine. However, those in the liver from G1 HO were decreased by 26%. Taurine levels in both the liver and brain were increased in mice whose drinking water was supplemented with 0.05% taurine (G3 HOT and G4 HOT). Taurine concentrations were not significantly different in the brain of HOT mice compared to WT. Although taurine concentrations in the liver from G3 HOT and G4 HOT increased significantly compared to G3 HO and G4 HO, they were not completely restored to WT levels. Although some G3 HO PD1 pups did not show a milk spot, taurine concentrations in the liver and brain were not significantly different in these two groups: offspring with a milk spot (G3 HO W/MS) versus offspring without a milk spot (G3 HO W/O MS) in the liver and brain, 2.1 ± 0.1 versus 1.8 ± 0.2 and 6.0 ± 0.5 versus 5.5 ± 0.5, respectively.

### 3.4. Gene Expression in Liver and Brain

We used a microarray of more than 9,000 genes to examine the effect of the CSAD knockout and taurine treatment on gene expression in PD1 HO mice (Figure 3). As expected, CSAD mRNA was not detected in CSAD KO. Three hundred genes in the liver and 145 genes in the brain were changed by more than 2-fold in the CSAD^−/−^ at PD1 (Table 4). Genes in the liver were mostly upregulated while the genes in the brain were mostly downregulated. Genes selected from microarray data were summarized in Table 5. Of the taurine-related genes, CSAD, CDO, ADO and TauT, only CSAD was markedly reduced confirming deletion of this gene. Antioxidant enzymes including Gpx1 and 3 and Prdx 2 and 3 were examined because neonatal death may be caused by oxidative stress. Uracil phopshorylase 2 and serine dehydratase, two metabolic enzymes, demonstrated significant upregulation and were further studied using *RT* ^2^ qPCR. Prolactin and lactoferrin were significantly down-regulated. Since these factors are intimately associated with production and function of milk and because milk spots were a predictor for survival, downregulation of these genes was confirmed using *RT* ^2^ qPCR. Taurine-related genes including CSAD, CDO, ADO, and TauT were examined using *RT* ^2^ qPCR (Table 6). The mRNA expression levels of TauT in the liver were not affected by taurine treatment while CDO was significantly increased in G3 HO compared to WT. The gene expression of ADO in G3 HOT was increased marginally. Expression of two antioxidant enzymes in the liver, peroxireductase 2 and 3 (Prdx 2 and Prdx 3), was not significantly different in CSAD^−/−^ compared to WT (Table 7). Prdx 2 in the brain from G3 HOT was significantly increased. However, glutathione peroxidase 1 and 3 (Gpx1 and Gpx3) were increased significantly in CSAD^−/−^ liver and restored to wild type levels in taurine-treated CSAD^−/−^ animals.

**Figure 5:**
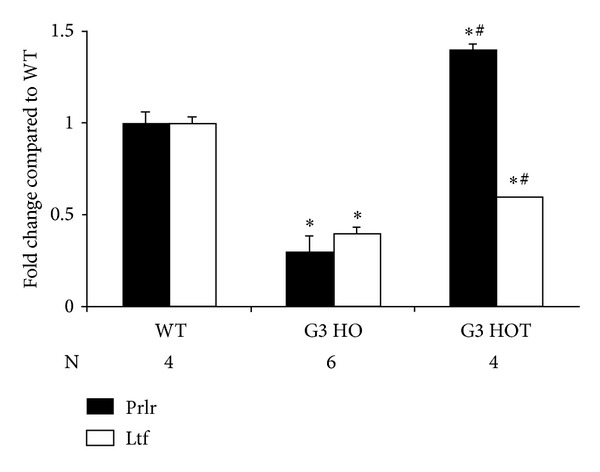
Fold changes of Prlr and Ltf in G3 HO and G3 HOT compared to those in WT. The liver and brain at PD 1 were homogenized with Trizol and mRNA was extracted using RNeasy kit. After cDNA was produced by reverse transcriptase, 10 ng of cDNA was mixed with SYBR master mix and reacted with primers using PCR system. Data are expressed as fold change compared to WT. (∗) is significantly different compared to WT, *P* < 0.001 and # is significantly different compared to G3 HO, *P* < 0.01.

Since newborn G3 CSAD^−/−^ often lacked milk spots, a predictor of survival in newborn mice, we examined the expression of lactoferrin (Ltf) and the prolactin receptor (Prlr) and found that their expression was significantly decreased in CSAD^−/−^ animals. Prlr expression in the liver was brought to levels higher than WT by taurine treatment, but expression of Ltf was not restored (Figure 5). Since the absence of a milk spot predicts mortality, we compared the gene expression of pups with a milk spot to those without a milk spot (Table 8). G3 HO pups without a milk spot (G3 HO w/o MS) demonstrated significantly higher expression of Cdo, Upp2, and Sds compared to those with a milk spot (G3 HO w/MS). However, Ltf was significantly decreased in G3 HO w/o MS compared to G3 HO w/MS. Expression of Sds in G3 HO w/MS was significantly increased compared to WT (Table 9). Expression of Cdo, Upp2, and Sds in G3 HOT was restored to that in WT.

## 4. Discussion

We have generated a new mouse model to study the physiological role of taurine by knocking out the CSAD gene. Disruption of the CSAD gene in this mouse was documented by Southern analysis [Figure 1(b)], the absence of CSAD mRNA and protein was shown by Northern and Western analyses [Figures 1(d) and 1(e)], and the taurine concentration in the plasma of CSAD^−/−^ animals was reduced by >80% (Table 2).

The physiological role of taurine has been studied in the cat [2], where it is an essential amino acid, and in the rat after treatment with guanidinoethanesulfonate (GES), a competitive inhibitor of taurine transport [29, 30]. More recently mouse models of taurine deficiency have been engineered to facilitate this investigation: two taurine transporter knockout mouse models (TauT KO) show reduced levels of taurine in various tissues in heart, brain, muscle, kidney, and liver [31–33]. These TauT KO models demonstrate developmental effects in various organs including the retina, liver, brain, muscle and heart. In addition taurine deficient mice were developed by knocking out the gene for CDO which produces cysteine sulfinic acid, a substrate for CSAD, from cysteine [34–36]. These mice have severe taurine deficiency and increased catabolism of cysteine to hydrogen sulfide, which leads to pulmonary and pancreatic toxicity.

The CSAD knockout mouse that we describe here shows drastically reduced endogenous taurine biosynthesis and complete dependency on exogenous taurine for normal reproductive performance (Table 3). Heterozygous CSAD KO mice had reduced levels of plasma taurine but CSAD haploinsufficiency had no detectable effect on reproduction. G1 CSAD^−/−^ derived from heterozygous parents had much reduced plasma levels of taurine (Table 2) but had litters of normal size that were indistinguishable from litters of WT or CSAD^+/−^ (Table 3). Offspring from G2 CSAD^−/−^ and G3 CSAD^−/−^, however, had very low survival rates (Table 3). Thus, abnormal reproductive performance appeared from the second generation of taurine deficient CSAD^−/−^ while reproduction of the first generation was not affected. Taurine supplementation in the drinking water (G2 HOT and G3 HOT) prior to, as well as during gestation and after birth, completely restored the pup survival rate. Reproduction performance was not significantly different between G2 HOT, G3 HOT, and WT, indicating that fetus and neonates were healthy when they were supplied with necessary taurine through the placenta and milk. These results strongly indicate that the reduced level of taurine may be responsible for the neonatal mortality. However, since reduced taurine is not 100% lethal and taurine concentrations in G3 HO w/MS and G3 HO w/o MS are not significantly different, there are apparently additional environmental or stochastic factors that influence neonatal mortality. The cross-fostering results indicate that newborn pups have difficulty with suckling. This suggests that the reduced taurine levels impact the suckling ability and it seems likely that this defect is associated with prenatal development.

These results are generally consistent with studies of taurine deficiency in the cat. Studies have shown that the reproductive performance of cats fed taurine deficient diets (0 or 0.01%) was poor and that they frequently had stillborn or low birth weight kittens with severe neurological abnormalities [2, 37, 38]. The offspring from taurine deficient cats showed abnormal births including stillbirths, abnormal brain and eye development, and poor growth. In contrast, G1 CSAD^−/−^ born from HT and with low taurine levels grew normally and their offspring also appeared normal (Table 3). A high rate of neonatal death occurred in most of the offspring from G2 CSAD^−/−^ and G3 CSAD^−/−^. These data indicate that low plasma taurine levels like G1 HO (Table 2) are not sufficient to induce poor reproductive performance. Taurine concentrations at PD1 in the liver and brain from G1 HO were similar to WT (Figures 4(a) and 4(b)). G1 HO with a taurine rich fetal environment and G2 HO with a taurine poor fetal environment implicate a taurine deficient fetal life for a higher death rate [Table 3 and Figures 4(a) and 4(b)]. In the cat model the offspring which show abnormalities or death develop in a low taurine environment due to intrinsically low levels of CDO and CSAD as well as the absence of dietary taurine.

To determine whether oxidants contributed to neonatal death, antioxidant enzymes including Gpx1 and 3 as well as Prdx 2 and 3 were examined [39–44]. Since Gpx1 and 3 were significantly increased, oxidants may be contributing factors in the neonatal death of HO mice (Table 7). These data support our hypothesis that taurine supplementation may restore oxidant induced damage.

We used microarray analysis to examine how the mRNA expression of more than 9,000 genes might have been altered by the deletion of CSAD. It is important to keep in mind that these changes could be due to transcriptional differences or differences in mRNA processing or mRNA stability which may or may not lead to more or less of the protein itself. Analysis of the brain and liver from WT and G3 HO at PD1 showed that gene expression in the liver was mostly increased while gene expression in the brain was mostly decreased (Table 4). Using *RT* ^2^ qPCR gene expression of relevant genes to fetal death was examined at PD1 in WT, G3 HO, and G3 HOT (Tables 6, 7, 8, and 9). As expected, CSAD was absent in the KO. *RT* ^2^ qPCR data in the levels of ADO, CDO, and CSAD are consistent in the brain and liver with data previously described [15, 17]. Taurine in the liver, brain, and blood of the CSAD^−/−^ mouse is not completely absent, presumably because of the alternative pathway for taurine biosynthesis through ADO (Table 2 and Figure 4) [2, 17]. The mRNA expression of ADO was not significantly different in both the liver and brain in G3 HO (Table 6) and taurine concentrations were markedly reduced in G2, G3, and G4 HO [Figures 4(a) and 4(b)], indicating that the alternative, ADO, pathway is a relatively minor source for taurine production. TauT was not changed in three groups including WT, G3 CSAD^−/−^, and G3 HOT, suggesting that the increase of taurine in G3 HOT is not mediated by the induction of TauT mRNA. These data also indicate that TauT does not show the increased expression at PD1 that is seen later in taurine deprived/deficient animals [36].

Because newborn G3 CSAD^−/−^ often lacked milk spots, which are a predictor of survival in newborn mice, we examined the gene expression of Ltf and Prlr which were markedly reduced in microarray analysis (Table 5). Ltf and Prlr in the liver were decreased significantly in G3 HO but only Prlr was restored by taurine supplementation (G3 HOT) (Figure 5). Ltf has innate immune function to protect newborn offspring from infection and is elevated in colostrum [45–47]. Prolactin is a lactogenic hormone and regulates the output of insulin-like growth factor-1. Genetic ablation of Prlr results in mice which show multiple defects in reproduction leading to infertility, altered maternal behavior, and reduced bone development [48–50]. The decreased expression of these two genes may contribute to CSAD^−/−^ neonatal death. Meanwhile, CDO was increased in G3 HO without milk spots but not in G3 HO with milk spots. These data indicate that increase of CDO in G3 HO is attributable to the absence of milk (Tables 6 and 8). Since uridine phosphorylase 2 (Upp2) and serine dehydratase (Sds) in G3 HO were dramatically increased in microarray analysis (Table 5), these genes were examined using *RT* ^2^ qPCR. Upp2 and Sds were significantly increased and Lft was significantly decreased in G3 HO without milk spots, compared to G3 HO with milk spots (Table 8). Upp 2 catalyzes the cleavage of uridine to uracil and ribose which are involved with energy supply and nucleotide synthesis [51–53]. The significance of the increase in Upp 2 in G3 HO without milk spot is unclear but may be a consequence of the morbidity of these mice. Serine is a substrate for Sds with production of pyruvate (precursor of glucose) and NH_3_ [54–56]. The activation of this enzyme is augmented by starvation [57]. The lack of a milk spot and subsequent death indicate starvation which is responsible for the large increase in Sds. Expression of Sds in G3 HO with milk spots was remarkably lower than in G3 HO without milk spots. However, expression of Sds in G3 HO with milk spots is significantly increased compared to WT, indicating that low taurine in G3 HO may induce gluconeogenesis by an increase in Sds. This result suggests that Sds expression is synergistically up-regulated by the HO genotype and by starvation/absence of a milk spot. WT expression levels of these genes were restored in G3 HOT, suggesting that taurine corrects abnormalities in nucleotide and amino acid metabolism due to an increase of Upp2 and Sds (Table 9). Ltf is widely present in fluids such as milk and colostrum with a high affinity for iron. Lft, a component of the innate immune system, is important in bacteriostasis and required for optimal neutrophil function. The significant decrease of Lft in G3 HO w/o MS is consistent with the importance of this protein for survival. Taurine concentrations in the brain, liver, and plasma from CSAD^−/−^ were significantly lower compared to WT and CSAD^+/−^. These data indicate that CSAD is an effective target for lowering taurine to symptomatic levels and that wild-type taurine levels can be restored in the brain and liver of CSAD KO mice by adding taurine to their drinking water. Taurine treatment dramatically increased survival rates of G3 HO and G4 HO mice. These data indicate that this CSAD KO mouse will be a powerful additional model for the analysis of the physiological role of taurine, particularly since taurine requirements can be supplied in food or water.

## Figures and Tables

**Table 1:** Sequences of PCR primers.

Primer	Sequence (5′-3′)
^1^ *β*-geo F	TTATCGATGAGCGTGGTGGTTATGC
*β*-geo R	GCGCGTACATCGGGCAAATAATATC
^ 2^VIS F	GCCTTGCCACAGGAGATTAT
VIS R	AACACGCAGACTCAGGAACA

^1^Primers for vector (pGT1xf).

^2^Primers for vector insertion site.

**Table 2:** Plasma taurine concentrations in CSAD KO.

Genotype (*N*)^1^	Taurine concentrations^2^
WT (7)	990.1 ± 95.3^3^
HT (6)	677.9 ± 106.8*
G1 HO (9)	163.6 ± 10.7**
G2 HO (8)	208.0 ± 42.3**

^1^Number of animals at 8 weaks of age. Two or three mice per each litter.

^2^ *μ*M.

^3^Data are expressed as mean ± SE.

*P < 0.05, compared to WT.

**P < 0.001, compared to WT.

**Table 3:** Survival rate of litters from mating pairs as well as average number of total and surviving pups per litter.

Mating pairs	WT	HT	G1 HO	G2 HO	G2 HOT^1^	G3 HO	G3 HOT^2^
Number of mating pairs	9	14	10	13	13	10	16
Average number of total pups per litter	5.1 ± 0.8^4^	6.2 ± 0.9	6.1 ± 0.7	4.5 ± 0.4	6.4 ± 0.6	4.9 ± 0.6	5.5 ± 0.5
Surviving litters from mating pairs^3^	9	14	10	2	12	1	15
Average number of surviving pups per litter	4.9 + 0.8	6.2 + 0.9	5.6 ± 0.6	0.9 ± 0.2*	5.4 ± 0.7	0.4 ± 0.3*	5.3 ± 0.5

^1^Mating pairs (G2 HO) were treated with taurine in drinking water (0.05%) 10 days prior to mating.

^2^Mating pairs born from G2 HOT and treated with taurine after weaning.

^3^Litters are designated as “surviving litter” if one or more pups grew to weaning.

^4^Mean ± SE.

*P < 0.002 compared to WT.

**Table 4:** Gene expression in G3 HO with more than 2-fold difference compared to WT.

	Total genes	Upregulation	Downregulation
Liver from G3 HO*	305	265	40
Brain from G3 HO	145	49	96

*Microarray was performed using two mice from G3 HO and WT at PD1.

**Table 5:** Fold change of genes in the liver of G3 HO compared to WT*.

	Fold change	Regulation
CSAD	9.6	Down
Gpx 1	1.5	Up
Gpx 3	3.3	Up
Prdx 2	1.4	Up
Prdx 3	1.2	Up
Prlr	2.7	Down
Ltf	2.4	Down
Upp 2	9.4	Up
Sds	6.9	Up

*Microarray was performed using two mice from G3 HO and WT at PD1.

**Table 6:** Fold change of taurine-related genes in G3 HO and G3 HOT compared to WT.

Gene	Genotype	Liver	Brain
Csad	WT	1.0 ± 0.03^1^	ND^2^
	G3 HO	<0.002**	
	G3 HOT	<0.001**	

Cdo	WT	1.0 ± 0.05	1.0 ± 0.03
	G3 HO	1.3 ± 0.10*	1.1 ± 0.10
	G3 HOT	1.1 ± 0.09	1.3 ± 0.30

Ado	WT	1.0 ± 0.03	1.0 ± 0.09
	G3 HO	1.1 ± 0.06	1.0 ± 0.10
	G3 HOT	1.3 ± 0.08*	1.1 ± 0.20

TauT	WT	1.0 ± 0.06	1.0 ± 0.08
	G3 HO	1.2 ± 0.07	1.4 ± 0.10
	G3 HOT	1.2 ± 0.09	1.3 ± 0.30

^1^Data represent mean ± SE from 4 WT, 6 G3 HO, and 4 G3 HOT at PD1, respectively.

^2^ND means not determined due to low expression in the brain.

*Significantly different compared to WT, *P* < 0.05.

**Significantly different compared to WT, *P* < 0.001.

**Table 7:** Fold change of antioxidant genes in G3 HO and G3 HOT compared to WT.

Gene	Genotype	Liver	Brain
Gpx 1	WT	1.0 ± 0.05	ND^#^
	G3 HO	1.5 ± 0.11*	
	G3 HOT	1.2 ± 0.09	

Gpx 3	WT	1.0 ± 0.14	1.0 ± 0.05
	G3 HO	2.5 ± 0.51*	1.1 ± 0.10
	G3 HOT	0.9 ± 0.08	1.2 ± 0.11

Prdx 2	WT	1.0 ± 0.05	1.0 ± 0.04
	G3 HO	1.6 ± 0.34	1.0 ± 0.08
	G3 HOT	1.0 ± 0.05	1.7 ± 0.89*

Prdx 3	WT	1.0 ± 0.10	ND
	G3 HO	1.1 ± 0.08	
	G3 HOT	1.0 ± 0.07	


^#^ND means not determined.

*Data are expressed as mean ± SE and significantly different, *P* < 0.05. Four mice were used in WT and G3 HOT and six mice at PD1 were used in G3 HO.

**Table 8:** Fold change of G3 HO with a milk spot and without a milk spot compared to WT in the liver.

	G3 HO w/o MS	G3 HO w/MS
Csad	0.001 ± 0.0**	0.003 ± 0.0
Cdo	1.6 ± 0.04*	1.1 ± 0.14
Ado	1.1 ± 0.07	1.3 ± 0.07
TauT	1.1 ± 0.08	1.4 ± 0.09
Gpx 1	1.7 ± 0.16	1.3 ± 0.12
Gpx 3	2.1 ± 0.35	2.9 ± 0.99
Prdx 2	2.2 ± 0.68	1.1 ± 0.07
Prdx 3	1.1 ± 0.15	1.0 ± 0.07
Prlr	0.2 ± 0.03	0.4 ± 0.14
Ltf	0.4 ± 0.02*	0.5 ± 0.03
Upp 2	8.1 ± 1.44*	1.9 ± 0.51
Sds	22.6 ± 6.64*	2.3 ± 0.42

*Significantly different (*P* < 0.05) between G3 HO w/o MS and G3 HO w/MS.

**Data represent mean ± SE from 3 mice of each group at PD1.

**Table 9:** Gene expression of Cdo, Upp 2, and Sds in the liver.

	Cdo	Upp 2	Sds
WT	1.0 ± 0.05^@^	1.0 ± 0.02	1.0 ± 0.22
G3 HO w/o MS	1.6 ± 0.04^∗#^	8.1 ± 1.44^∗#^	22.6 ± 6.64^∗#^
G3 HO w/MS	1.1 ± 0.14	1.9 ± 0.51	2.3 ± 0.42*
G3 HOT	1.1 ± 0.09	0.9 ± 0.10	1.2 ± 0.10

*Significantly different, *P* < 0.05 compared to WT.

^#^Significantly different, *P* < 0.05 compared to G3 HO w/MS.

^@^Data represent mean ± SE. Four mice were used in WT and G3 HOT. Three mice were used in G3 HO w/o MS and G3 HO w/MS.

## Abbreviations

CSAD: Cysteine sulfinic acid decarboxylase
CSAD KO: Cysteine sulfinic acid decarboxylase knockout mice
CDO: Cysteine dioxygenase
CDO KO: Cysteine dioxygenase knockout mice
ADO: Cysteamine(2-aminoethanethiol) dioxygenase
WT: Wild type (CSAD^+/+^)
HT: Heterozygotic mice (CSAD^+/−^)
HO: Homozygotic mice (CSAD^−/−^)
HOT: Homozygotic mice treated with 0.05% taurine
TauT: Taurine transporter
TauT KO: Taurine transporter knockout mice.
Gpx 1: Glutathione peroxidase 1
Gpx 3: Glutathione peroxidase 3
Prdx 2: Peroxireductase 2
Prdx 3: Peroxireductase 3
Prlr: Prolactin receptor
Ltf: Lactoferrin
Upp 2: Uridine phosphorylase 2
Sds: Serine dehydratase
G1, G2, G3, G4: Generations 1, 2, 3, and 4
G1 HO: HO (CSAD^−/−^) mice born from HT (CSAD^+/−^) parents
G2 or G3 HO: Mice born from G1 HO parents or G2 HO
G3 HO w/MS: The third generation of homozygotes with a milk spot
G3 HO w/o MS: The third generation of homozygotes without a milk spot
PD1: Postnatal Day 1
PITC: Phenyl isothiocyanate
HPLC: High Performance Liquid Chromatography
GES: Guanidinoethanesulfonate.
